# Supplementary material for: WordCommentsAnalyzer: A windows software tool for qualitative research
Source: F1000Res. 2018 Sep 4;7:536. Originally published 2018 May 3. [Version 2] doi: 10.12688/f1000research.14819.2 (PMC6137411; doi:10.12688/f1000research.14819.2)
Supplement: Supplementary file 3 [file f1000research-7-17614-s0002.tgz › b2a79496-4ada-47c4-9682-a99ce867836d.zip!/Eight Iranian Health Journals/Archives of Rehabilitation_29.docx]

© Archives of Rehabilitation. This work is licensed under CC BY-NC 4.0 (https://creativecommons.org/licenses/by-nc/4.0/).

Volume & Issue: دوره 12 -

Title: بررسی تأثیر متون آموزشی تهیه شده در زمینه اعتیاد و عوارض آن بر میزان آگاهی دانش آموزان پسر مقاطع مختلف تحصیلی ‌شهر تهران

http://rehabilitationj.uswr.ac.ir/article-1-1070-fa.html

هدف: هدف از این مطالعه تعیین تأثیر بسته‌های آموزشی تهیه شده بر میزان آگاهی دانش‌آموزان مقاطع مختلف تحصیلی شهر تهران در زمینه اعتیاد و عوارض آن می‌باشد. روش بررسی: این مطالعه شبه تجربی از طریق روش نمونه‌گیری خوشه‌ای چند مرحله‌ای بر روی نمونه ۱۶۳ نفری از دانش‌آموزان مقاطع مختلف تحصیلی منطقه یک تهران، با استفاده از پرسشنامه محقق ساخته انجام پذیرفت. پرسشنامه‌های آزمون به صورت پیش آزمون (قبل از مطالعه بسته‌های آموزشی) بین دانش آموزان کلیه مقاطع تحصیلی توزیع و جمع آوری گردید و سپس پرسشنامه‌های پس آزمون (بعد از مطالعه بسته‌های آموزشی) که حاوی سئوالات باز پاسخ و سه سئوال بسته پاسخ (چهارگزینه‌ای) بود که به فاصله یک هفته (با توجه به حجم مطالب بسته‌های آموزشی) بین دانش‌آموزان توزیع و جمع آوری شد و با استفاده از آزمون تی زوجی مورد تجزیه و تحلیل قرار گرفت. یافته‌ها: یافته‌های این مطالعه بیانگر این است که میانگین نمره آگاهی قبل (۷/۶۱) و بعد از مداخله (۹/۴۴) در هر سه مقطع تحصیلی ابتدایی (به جز کلاس چهارم و پنجم)، راهنمایی و متوسطه معنادار بوده است. نتیجه‌گیری: با توجه به نتایج مطالعه می‌توان چنین عنوان کرد که آموزش، بر افزایش سطح آگاهی دانش آموزان مقاطع مختلف تحصیلی در زمینه اعتیاد و عوارض آن مؤثر بوده است.

Title: مقایسه ویژگی‌های شخصیتی مردان وابسته به مواد افیونی و مردان وابسته به مت‌آمفتامین

http://rehabilitationj.uswr.ac.ir/article-1-993-fa.html

هدف: پژوهش حاضر با هدف مقایسه ویژگی‌های شخصیتی مردان وابسته به مواد افیونی و مردان وابسته به مت‌آمفتامین انجام شده است.  روش بررسی: پژوهش حاضر از نوع علی مقایسه‌ای و مقطعی می‌باشد. در این مطالعه با توجه به ملاک‌های ورود، 80 نفر از افراد وابسته به مواد افیونی و مت‌آمفتامین از بین کلیه افراد مراجعه کننده به مرکز تولدی دوباره به صورت نمونه‌گیری قضاوتی انتخاب و بر اساس متغیرهای جمعیت‌شناختی همتا شدند. برای گردآوری داده‌ها، پرسشنامه دموگرافیک و پرسشنامه ویژگی‌های شخصیتی نئو (فرم کوتاه) (NEO-FFI-R) مورد استفاده قرار گرفت. داده‌ها با استفاده از آزمون تی مستقل تحلیل شدند. یافته‌ها: یافته‌ها نشان داد که بین میانگین نمرات دو گروه از مردان وابسته به مواد افیونی و مردان وابسته به مت‌آمفتامین در حیطه روان‌رنجوری (N)، خوشایندی (A) و وظیفه‌شناسی (C) پرسشنامه شخصیتی نئو تفاوت معنادار وجود دارد (0/05>P). بدین معنا که سطح روان‌رنجوری در مردان وابسته به مواد افیونی در مقایسه با مردان ابسته به مت‌آمفتامین بالاتر و سطح خوشایندی و وظیفه شناسی در مردان وابسته به مت‌آمفتامین در مقایسه با مردان وابسته به مواد افیونی بالاتر است. هر دو گروه به لحاظ میزان برونگرایی (E) و گشودگی (O) از وضعیت مشابهی برخوردار بودند (P>0/05) نتیجه‌گیری: با توجه به یافته‌های پژوهش، بین ویژگی شخصیتی مردان وابسته به مواد افیونی و مت‌آمفتامین تفاوت وجود دارد. بنابراین شناخت شخصیت و ویژگی‌های آن می‌تواند در پیشگیری و درمان مؤثر باشد.

Title: بررسی اثربخشی آموزش مهارت‌های زندگی بر تغییر نگرش نسبت به مواد مخدر در میان سربازان وظیفه شاغل در زندان‌های استان تهران

http://rehabilitationj.uswr.ac.ir/article-1-805-fa.html

هدف: این پژوهش با هدف بررسی اثربخشی آموزش‌های مهارت زندگی بر تغییر نگرش، نسبت به مواد مخدر در میان سربازان وظیفه شاغل در زندان‌های استان تهران در سال 1389 انجام گردید. روش بررسی: در این پژوهش مداخله‌ای، سه گروه شامل یک گروه آزمایش و دو گروه گواه (درونی و بیرونی)، شامل ۱۹۶ نفر از سربازان وظیفه مشغول به خدمت در زندان‌های استان تهران، که با روش تصادفی نظام‌دار (سیستماتیک) انتخاب شده بودند، شرکت داشتند. گروه مداخله آموزش‌ها را در سیزده جلسه دریافت کرد؛ پرسشنامه‌ها در سه مرحله شروع، دو ماه بعد از شروع و شش ماه بعد از شروع تکمیل شد. ابزار جمع آوری داده‌ها شامل یک پرسشنامه دموگرافیک محقق ساخته و یک پرسشنامه 10 عاملی نگرش نسبت به اعتیاد بود. روایی و پایایی پرسشنامه نگرش سنج به ترتیب 0/90 و 0/91 بود. برای تجزیه و تحلیل داده‌ها از آزمون‌های تی زوجی و تی مستقل و تحلیل واریانس تک‌راهه و آزمون‌های تعقیبی توکی استفاده شد. یافته‌ها: سربازان برگزیده در سه گروه آموزش (65 نفر)، گروه گواه درونی (64 نفر) و گروه گواه بیرونی (67 نفر) قرار گرفتند. مقایسه این سه گروه باهم نشان داد نگرش کلی به اعتیاد (0/001>P)، نگرش نسبت به مصرف مواد مخدر (0/001>P)، نگرش نسبت به علت مصرف مواد مخدر (0/001>P) نگرش نسبت به شخصیت فرد معتاد (0/004=P)، نگرش اجتماع نسبت به اعتیاد (0/001>P)، نگرش نسبت به ترک اعتیاد (0/001>P)، نگرش نسبت به گریز از اعتیاد (0/011=P) و نگرش نسبت به اعتیاد به عنوان راه حل در زندگی (0/007=P)، بهبود یافته و از نظر آماری معنادار بوده است. نتیجه‌گیری: نتایج نشان داد که مداخلات آموزش مهارت زندگی بر تغییر و بهبود نگرش در میان سربازان آموزش‌دیده مؤثر است و می‌تواند نقش مهمی در کاهش سوء مصرف مواد مخدر در جامعه ایفا نماید.

Title: نگاهی اجمالی بر وضعیت پژوهش‌های سوء مصرف و وابستگی به مواد در ایران

http://rehabilitationj.uswr.ac.ir/article-1-977-fa.html

هدف: این مطالعه با هدف بررسی وضعیت پژوهش‌های انجام شده در خصوص سوء مصرف و وابستگی به مواد در ایران انجام شده است. روش بررسی: در این مطالعه مروری، کلیه اطلاعات مرتبط در دسترس، از جمله پژوهش‌ها، مصاحبه‌های انجام شده، سایت‌های مرتبط، گزارشات، دستورالعمل‌ها، اظهارنظر مسئولان، قوانین، و غیره مورد استفاده قرار گرفته شده است. یافته‌ها: اعتیاد یک مشکل عمده در کشور ما است که عوارض شدید و فوریت در مداخله را ایجاب می‌نماید. به دلیل سختی و طولانی بودن پژوهش‌های پیشگیری، مطالعات پیشگیری و اجتماعی در مورد اعتیاد ناچیز هستند، تعداد قابل‌توجهی از تحقیقات در حوزه درمان اعتیاد، در کشور ما صورت گرفته است. با توجه به حادثه‌خیز بودن کشور و نقش موارد تنش‌زایی مثل زلزله، سیل و… در مصرف مواد، اکثر تلاش‌ها بر کنترل مخدرها متمرکز است که نشان از پیش‌بینی ضعیف آینده و لزوم پایش مداوم تغییرات نوع ماده مصرفی و آینده‌نگری می‌باشد. شیوع استفاده تزریقی در مقایسه با غیرتزریقی افزایش یافته است و ۱۵ تا ۲۳ درصد مصرف‌کنندگان تزریقی در ایران آلوده به HIV هستند. در بیشتر مطالعات، هزینه و مخارج مستقیم درمان اعتیاد لحاظ می‌شود و مواردی مثل هزینه مبارزه، پیشگیری، قاچاق مواد و… مدنظر قرار نمی‌گیرد. نتیجه‌گیری: با توجه به شرایط مساعد نگرشی در کل جامعه، و قوانین و دستورالعمل‌های مرتبط با آن، لازم است که برنامه‌ریزی مناسب و جامعی در زمینه پژوهش‌های پیشگیری و اجرایی علاوه بر درمان در کشور صورت گیرد.

Title: شناسایی طرح‌واره‌های ناسازگار اولیه در افراد وابسته به مواد افیونی و افراد غیروابسته

http://rehabilitationj.uswr.ac.ir/article-1-972-fa.html

هدف: وابستگی دارویی پدیده پیچیده‌ای است که از تعامل چندین عامل ناشی می‌شود و محققین به دنبال حل این مسئله از رویکردهای مختلف استفاده می‌کنند پژوهش حاضر با هدف شناسایی طرح‌واره‌های ناسازگار اولیه در افراد وابسته به مواد افیونی در نظر دارد تسهیل کننده مسیر درمانی این افراد باشد. روش بررسی: این پژوهش یک مطالعه میدانی و از نوع پس رویدادی می‌باشد. جامعه آماری کلیه افرادی را در بر می‌گیرد که جهت ترک اعتیاد به کلینیک آبان در تهران مراجعه نموده‌اند. حجم نمونه 60 نفر است. تعداد 30 نفر به روش نمونه‌گیری تصادفی ساده از لیست مراجعین انتخاب شد و تعداد 30 نفر نیز از بین بستگان این افراد به عنوان گروه گواه در نظر گرفته شد. پرسشنامه‌های مشخصات دمو‌گرافی و طرح‌واره یانگ ، بر روی هر دو گروه اجرا گردید. یافته‌ها: با کمک نرم افزار SPSS و با استفاده از آزمون تی مستقل مشخص گردید که در حوزه بریدگی/طرد، خودگردانی و عملکرد مختل (0/001>P) اختلاف معناداری بین دو گروه وجود دارد همچنین در حوزه جهت‌مندی نیز اختلاف معنادار بود (0/005>P).در حوزه محدودیت‌های مختل و گوش به زنگی بیش از حد و بازداری این اختلاف معنادار نبود. ولی به طور کلی در کلیه حوزه‌ها طرح‌و‌اره‌های افراد وابسته به مواد افیونی از ناکارآمدی بیشتری برخوردار بودند.  نتیجه‌گیری: از آنجایی تکنیک طرح‌واره درمانی توسط روانشناسانی که در مراکز ترک اعتیاد فعالیت دارند می‌تواند روشی کارآمد محسوب گردد لذا شناخت طرح‌واره‌های ناسازگار اولیه در افراد وابسته به مواد افیونی می‌تواند مسیر درمان را کوتاه نماید.

Title: نیازسنجی آموزشی مرتبط با رشته‌های بلندمدت دانشگاهی اعتیاد در ایران

http://rehabilitationj.uswr.ac.ir/article-1-1067-fa.html

هدف: کارشناسان حیطه پیشگیری و درمان مواد، در نظام مبارزه با مواد، تأثیر فراوانی بر اثر بخشی و عملکرد مجموعه نظام مقابله با سوء‌مصرف مواد دارند. از این رو، اجرای پژوهش‌هایی با هدف نیازسنجی آموزشی این افراد ضرورت می‌یابد. روش بررسی:مطالعه حاضر شامل دو بخش بود. بخش نخست بحث متمرکز گروهی متشکل از مسئولین ستادی حیطه مواد مخدر، کارشناسان دارای تجربه و مسئولین دانشگاه‌ها بود. همچنین با استفاده از پرسشنامه، دیدگاه ۵۲ نفر کارشناس در حیطه پیشگیری و درمان مصرف مواد مورد سنجش و ارزیابی قرار گرفت. اعضای جامعه تحقیق به روش تمام شماری از کارشناسان سازمان بهزیستی استان تهران گزینش شدند. یافته‌ها: از بین 45 نفر، 30 نفر (٪66) خواهان برگزاری دوره‌های آکادمیک در حیطه اعتیاد بودند. 19 نفر (٪42/2) خواهان برگزاری دوره‌های آموزشی کوتاه مدت از قبیل کارگاه‌های آموزشی بودند. بیشترین نیازهای آموزشی به ترتیب مربوط به بخش‌های درمان، مشاوره، کلیات، پیشگیری، پژوهش و کاهش آسیب به ترتیب در 41 (٪78/8)، 32 (٪61/5)، 30 (٪57/7)، 26 (٪50)، 22 (٪42/3) و ۵ نفر (٪9/6) گزارش شد و 34 نفر (٪65/4) علیرغم موارد فوق در سایر موارد متفرقه نیازهای آموزشی را گزارش نمودند. نتیجه‌گیری: با توجه به یافته‌های مطالعه حاضر که ٪۶۶ افراد خواهان راه اندازی یک دوره آکادمیک سوء مصرف مواد در دانشگاه‌های کشور می‌باشند، و فراوان ترین نیاز آموزشی جمعیت مورد مطالعه حاضر در حیطه درمان (٪78/8) و پس از آن مشاوره (٪61/5) می‌باشد.

Title: بررسی هزینه فایده سم‌زدایی معتادان بالای 40 سال مراجعه‌کننده به انجمن خیریه احیاء انسانی (کنگره 60)

http://rehabilitationj.uswr.ac.ir/article-1-1068-fa.html

هدف: در طی دو دهه اخیر فعالیت‌های مربوط به کاهش تقاضا و به ویژه درمان و باز توانی معتادان در ایران ارتقاء چشمگیری یافته است. هم اکنون آلترناتیوهای متعدد درمانی از جمله مراکز درمان سرپایی و بستری، روش‌های پرهیز مدار و ایمان مدار از جمله معتادان گمنام و مراکز خیریه تولدی دوباره به صورت دارویی و غیر دارویی فعالیت می‌کنند. روش سم زدایی و درمان در جمعیت خیریه احیاء انسانی (کنگره 60) به صورت انحصاری بوده و با جایگزینی شربت تریاک و کاهش تدریجی آن (طی 11 ماه) و همچنین گروه درمانی و معنویت درمانی می‌باشد. روش بررسی: پژوهش مذکور مطالعه‌ای اقتصادی و توصیفی است که در مدت 3 ماه (بدو ورود، یک ماه و سه ماه بعد درمان) به صورت طولی و هم‌گروهی انجام شده است. افراد در تحقیق شامل کلیه معتادان بالای 40 سالی است، که به انجمن (کنگره 60) جهت سم زدایی مراجعه نموده‌اند. انتخاب نمونه‌ها به روش نمونه گیری در دسترس بوده است. ابزار گردآوری اطلاعات پرسشنامه محقق ساخته جمعیت شناختی، و چک لیست پرداخت هزینه‌های درمانی (پرداخت مستقیم و غیر مستقیم) بوده است، داده‌ها با نرم افزار SPSS مورد تجزیه و تحلیل قرار گرفته‌اند.  یافته‌ها: از مجموع 33 نفر، 15 نفر بالای 50 سال بوده‌اند. تریاک با فراونی 12 نفر (٪36/4)، کراک و هروئین 14 نفر (٪42/3)، بالاترین درصد شیوع را داشتند. میانگین ماهیانه هزینه سوءمصرف مواد برای هر نفر 159849تومان می‌باشد. مجموع هزینه‌های اداری حدود 7720000 تومان در سال می‌باشد. سازمان‌های دولتی حمایت‌گر در حوزه اعتیاد و همچنین خیرین خصوصی حدود 35000000 تومان در سال 1389 به انجمن جهت تداوم خدمت رسانی کمک نموده‌اند که صرف باز توانی معتادان شده است. نتیجه گیری: در بررسی هزینه فایده و با توجه به کسر نهایی ٪58 و مبلغ خالص 16774474 تومان پس انداز شده، می‌توان نتیجه گرفت که اقدامات درمانی و سم زدایی انجمن فوق‌الذکر از اثر بخشی قابل ملاحظه‌ای برخوردار بوده است.

Title: مقایسه تلقین‌پذیری در افراد وابسته به مواد افیونی و افراد عادی

http://rehabilitationj.uswr.ac.ir/article-1-1069-fa.html

هدف: پژوهش حاضر با هدف مقایسه تلقین پذیری افراد وابسته به مواد افیونی و افراد عادی انجام گرفت. روش بررسی: در این پژوهش 76 فرد وابسته به مواد افیونی با روش نمونه گیری تصادفی نظامدار و 100 فرد عادی با روش نمونه گیری در دسترس انتخاب شدند، و با استفاده از آزمون ویژگی‌های شخصیتی حمزه گنجی مورد بررسی قرار گرفتند. جهت تحلیل داده‌ها از آزمونهای آماری تی مستقل و تحلیل واریانس استفاده شد. یافته‌ها: بین دو گروه عادی و وابسته به مواد افیونی در تلقین پذیری، تفاوت معنادار وجود دارد (0/05>P)، و نتایج تحلیل واریانس نشان داد که نوع ماده افیونی مصرفی، بر تلقین پذیری افراد وابسته به مواد افیونی تأثیر معناداری ندارد. همچنین در گروه افراد وابسته به مواد افیونی از لحاظ تلقین پذیری، بین زنان و مردان تفاوتی معناداری دیده نشد (0/05>P). نتیجه‌گیری: گروه افراد وابسته به مواد افیونی صرف نظر از نوع ماده افیونی مصرفی، در مقایسه با افراد عادی تلقین پذیری بیشتری دارند.

Title: تأثیر درمان نگهدارنده با متادون بر عملکرد جنسی مردان وابسته به مواد افیونی

http://rehabilitationj.uswr.ac.ir/article-1-851-fa.html

هدف: مطالعات نشان می‌دهد که اثرات درمان دارویی اعتیاد به مواد افیونی بر عملکرد جنسی متفاوت است، هدف پژوهش حاضر شناسایی اثر درمان نگهدارنده با متادون بر عملکرد جنسی مردان وابسته به مواد افیونی مراجعه کننده به مراکز درمانی ترک اعتیاد است. روش بررسی: مطالعه حاضر به صورت شبه تجربی انجام شد. جامعه آماری پژوهش حاضر را مردان متأهل وابسته به مواد تحت درمان نگهدارنده با متادون تشکیل می‌دادند. حجم نمونه 69 نفر برآورد و از طریق نمونه گیری در دسترس انتخاب شدند. ابزار مورد استفاده در پژوهش، پرسشنامه ارزیابی عملکرد جنسی بود که داده های پژوهش پس از تعیین اعتبار و پایایی آن در سه مقطع زمانی آغاز مطالعه، یک و سه ماه پس از درمان به صورت خود گزارش دهی جمع آوری گردیدند. تجزیه و تحلیل داده‌ها با استفاده SPSS نسخه 15 انجام پذیرفت.   یافته‌ها: نتایج پژوهش نشان داد که عملکرد جنسی و عملکرد جنسی(تمایل) واحدهای مورد پژوهش پس از درمان تفاوت معناداری با قبل از آن داشته است (0/001>P). به طوریکه پس از درمان عملکرد جنسی آن‌ها افزایش یافته بود و عملکرد جنسی (تمایل) آن‌ها کاهش یافته بود. همچنین نتایج حاکی از آن بود که هیچ تفاوت معناداری در عملکرد جنسی(نعوظ و ارگاسم و انزال) واحدهای مورد پژوهش مشاهده نشده است. نتیجه‌گیری: درمان نگهدارنده با متادون در عملکرد جنسی افراد وابسته به مواد افیونی مؤثر می‌باشد.

Title: بررسی ارتباط عوامل فردی ترک مواد مخدر با موفقیت سم‌زدایی

http://rehabilitationj.uswr.ac.ir/article-1-1071-fa.html

هدف: با توجه به تغییر الگوی مصرف مواد هدف این مطالعه ارزیابی مجدد عوامل فردی موثر بر موفقیت سم زدایی است. روش بررسی: در این مطالعه توصیفی تحلیلی، در مرحله جمع آوری داده‌ها مراکز خود معرف دولتی وابسته به سازمان بهزیستی شهر تهران و مراکز فعال خصوصی (به ترتیب با عملکرد کیفی ضعیف، متوسط و قوی بر اساس نظر کارشناسان ارزشیابی بهزیستی استان) ارزیابی شدند و مشخصات دموگرافیک، اطلاعات فردی، اطلاعات مربوط سیر درمان از 1372 نفر پرونده تکمیل شد. این داده‌ها با استفاده از آزمون رگرسیون لجستیک چندگانه تحلیل گردید. یافته‌ها: نتایج این مطالعه نشان داد که 258 نفر (%18/8) موفق به سم زدایی مواد مخدر گردیدند. مدل نهایی رگرسیون چندگانه نشان داد که مصرف‌کنندگان کراک 1/49 برابر در سم زدایی ناموفق‌تر بودند (0/05=P). مصرف‌کنندگان روزانه یک بار یا کمتر 2/4 برابر نسبت مصرف‌کنندگان روزانه 4 بار یا بیشتر در سم زدایی موفق‌تر بودند (0/01=P). همچنین کسانی که اطرافیان‌شان مواد مخدر مصرف نمی‌کردند 1/8 برابر کسانی که اطرافیان‌شان مواد مصرف می‌کردند (0/01>P) و مراجعه به علل عوارض روانی 1/94 برابر نسبت به مراجعه به عللی غیر از عوارض جسمی یا روانی در سم زدایی موفق تر بودند (0/01>P). نتیجه گیری: تغییر الگوی سوء مصرف مواد، افزایش شدت و عمق اعتیاد و عوامل فردی محیطی به عنوان عوامل تأثیر گذار بر موفقیت سم زدایی باید در امتداد ارتقای کیفی ارائه خدمات درمانی مورد توجه ویژه قرار گیرد.
